# Profiling individuals living with hypertension in Saudi Arabia 2021: a nationwide descriptive study

**DOI:** 10.3389/fcvm.2025.1579109

**Published:** 2026-01-02

**Authors:** Mohammed Senitan, Nora A. Althumiri, Zaied Alkhamaali, Ahmed M. Shaman, Nasser F. BinDhim

**Affiliations:** 1 Department of Public Health, College of Health Sciences, Saudi Electronic University, Riyadh, Saudi Arabia; 2 Informed Decision-Making for Research and Studies, Riyadh, Saudi Arabia; 3 Sharik Association for Research and Studies, Riyadh, Saudi Arabia; 4 Department of Clinical Pharmacy, College of Pharmacy, King Saud University, Riyadh, Saudi Arabia

**Keywords:** hypertension, sociodemographic determinants, comorbidities, cardiovascular risk, public health strategies

## Abstract

**Background:**

Hypertension is a major global health challenge affecting 1.3 billion adults, increasing risks of cardiovascular diseases, strokes, and kidney failure. Using data from a nationally representative surveillance system in Saudi Arabia, this study profiles adults living with treated or aware hypertension vs. the rest of the population and examines co-occurring sociodemographic, behavioral, and clinical factors in 2021.

**Methods:**

We analyzed data from the 2021 Sharik Health Indicators Surveillance System (SHISS), a nationwide cross-sectional phone survey employing proportional quota sampling across all 13 regions (*n* = 14,007; response rate = 68.3%). Hypertension status was defined as self-reported physician diagnosis with current treatment; blood pressure was not directly measured, and the survey relied entirely on self-report. Weighted descriptive statistics summarized prevalence, and multivariable logistic regression estimated adjusted odds ratios (AORs, 95% CIs) for factors associated with hypertension. The mean participant age was 36.7 ± 13.7 years.

**Results:**

Overall, 12.3% of respondents reported treated/aware hypertension. Among these, common comorbidities included hypercholesterolemia (47.9%), diabetes (42.8%), overweight/obesity (75.2%), heart disease (20.2%), and elevated depression risk (23.8%). In adjusted models, higher age (AOR 1.06 per year, 95% CI 1.05–1.06), education below bachelor's (AOR 1.31, 1.14–1.50), smoking (daily AOR 1.23, 1.03–1.48; occasional AOR 1.64, 1.28–2.08), overweight (AOR 1.93, 1.20–3.11), and obesity (AOR 2.53, 1.57–4.08) were significantly associated with hypertension. Strong associations were also observed with hypercholesterolemia (AOR 4.09, 3.56–4.71), heart disease (AOR 3.54, 2.84–4.42), and diabetes (AOR 2.64, 2.28–3.05).

**Conclusions:**

Adults aware of and treated for hypertension in Saudi Arabia exhibit a high burden of behavioral risks and multimorbidity. These findings can guide targeted primary care screening, integrated chronic disease management, and population-level risk reduction programs (e.g., smoking cessation, weight control), with priority to older adults, individuals with lower educational attainment, and those with co-existing diabetes or dyslipidemia.

## 1 Introduction

Hypertension (HTN), or high blood pressure, is one of the most prevalent non-communicable diseases worldwide and a leading contributor to cardiovascular morbidity and premature mortality. Globally, an estimated 1.3 billion adults are affected, with nearly two-thirds residing in low- and middle-income countries. Despite medical advances, fewer than half of those affected are diagnosed or treated, and only about one-fifth achieve adequate control, making HTN a persistent global health challenge and a priority in the World Health Organization's 2030 non-communicable disease (NCD) reduction goals (1, 2).

In Saudi Arabia, the burden of hypertension remains considerable. A 2023 national survey reported a 9.2% prevalence among individuals aged ≥15 years, rising steeply with age to exceed 50% among adults ≥65 years, and varying across regions from 6% in Najran to 10% in Makkah (3). A recent meta-analysis of 29 studies (*n* = 278,873) estimated a pooled prevalence of 22.7%, with awareness, treatment, and control rates of 42.8%, 59.4%, and 35%, respectively (4). These findings underscore persistent care gaps and highlight the need for improved detection and management strategies within the Saudi population (3–7).

Uncontrolled hypertension substantially elevates the risk of cardiovascular diseases, stroke, and renal failure. Comorbid conditions such as diabetes, obesity, and hypercholesterolemia and behavioral risk factors, particularly smoking and physical inactivity, further compound these outcomes (2, 8). Integrating behavioral and clinical interventions is therefore critical to effective prevention and control (9–13).

Although multiple studies have examined hypertension prevalence, few have provided a unified national profile that concurrently describes sociodemographic, behavioral, and clinical characteristics of individuals living with hypertension in Saudi Arabia. Most available data focus on isolated determinants or regional samples, limiting their usefulness for national policy and program planning.

Given the high prevalence and significant risks associated with HTN, it is critical to understand the current situation of adults living with HTN in Saudi Arabia. Comparing hypertensive individuals with the non-hypertensive population can provide insights into the impact of HTN on public health and help in developing targeted interventions. Such studies are vital for informing healthcare policies and strategies to manage and reduce the burden of HTN in Saudi Arabia (8, 14, 15). There remains a clear knowledge gap in generating a comprehensive national profile of adults living with HTN that integrates these dimensions within a single dataset.

This study seeks to address that gap. Using 2021 data from the Sharik Health Indicators Surveillance System (SHISS), we aim to profile adults living with HTN in Saudi Arabia and compare them with adults without HTN. By examining sociodemographic determinants, behavioral factors, and associated chronic diseases, this study provides novel insights to inform public health strategies, guide integrated management of non-communicable diseases, and support national healthcare policies.

## 2 Materials and methods

### 2.1 Design

This project involves a secondary analysis of data from the Sharik Health Indicators Surveillance System (SHISS) covering the first and second quarters of 2021. SHISS conducts brief, cross-sectional phone interviews across all 13 administrative regions of Saudi Arabia on a quarterly basis. Each interview lasts approximately 8–12 min and is carried out by a trained data collector. SHISS utilizes the ZdataCloud® research data collection system (8), which includes integrated eligibility and sampling modules to ensure sample distribution control and prevent human-related sampling bias. All data are coded and stored in the ZdataCloud® database (14–16). This study was reviewed and approved by the Sharik Association for Health Research Ethics Committee in accordance with national research ethics regulations (Approval No. 2021-2).

### 2.2 Definition of hypertension and other variables

Hypertension status was based on self-report of a physician diagnosis and current treatment (on-treatment diagnosis), as captured in the SHISS questionnaire. No direct blood pressure measurements were collected. Other chronic diseases (diabetes, hypercholesterolemia, heart disease, stroke, cancer, and chronic respiratory disease) were also self-reported based on physician diagnosis and treatment. Behavioral risk factors included cigarette, waterpipe, and e-cigarette smoking, physical activity, and fruit and vegetable consumption, following WHO and CDC frameworks. Obesity was calculated from self-reported height and weight using CDC BMI categories. Depression risk was screened using the PHQ-2 (cutoff ≥3).

### 2.3 Sample and sample size

Inclusion criteria were Saudi residents aged 18 years or older, Arabic speaking, and able to provide informed consent. Exclusion criteria were non-residents, individuals younger than 18, and those unable to complete the interview. SHISS employs a proportional quota sampling method to achieve an equal distribution of participants, stratified by age and gender, within and across the 13 administrative regions of Saudi Arabia (16). SHISS employs proportional quota sampling to ensure equal distribution by age group (18–36, ≥37 years) and gender within each region, resulting in 52 strata. Each quota required at least 134 participants per stratum (536 per region; ∼6,968 per wave). The technical automation of quota closure was handled by the ZdataCloud® system to ensure balanced demographic representation. Because multiple data collectors may complete calls simultaneously, oversampling can occur when two eligible participants finish interviews just before quota closure and is not detailed further here, to avoid distracting from the main study focus (14, 16–18).

### 2.4 Participants and recruitment

Participant recruitment was restricted to Arabic-speaking Saudi residents aged 18 and older. A random list of phone numbers was generated from the Sharik Association for Research and studies to identify potential participants. The Sharik database comprises individuals interested in future research projects and includes over 74,000 registered participants—at the data collection time—distributed across Saudi Arabia's 13 regions. Participants were phoned up to three times. If they did not respond, a new number with similar demographic characteristics was selected from the database until the quota was filled and automatically closed. After obtaining consent, the interviewer evaluated the participant's eligibility according to the quota completion criteria. Because the ZdataCloud® system automatically closes the quota once the target sample is reached, multiple phone calls occurring simultaneously may cause more than one participant to meet the eligibility requirements, potentially resulting in a slightly larger sample size for some quotas (16).

### 2.5 Questionnaire design and validation

**Figure 1:**
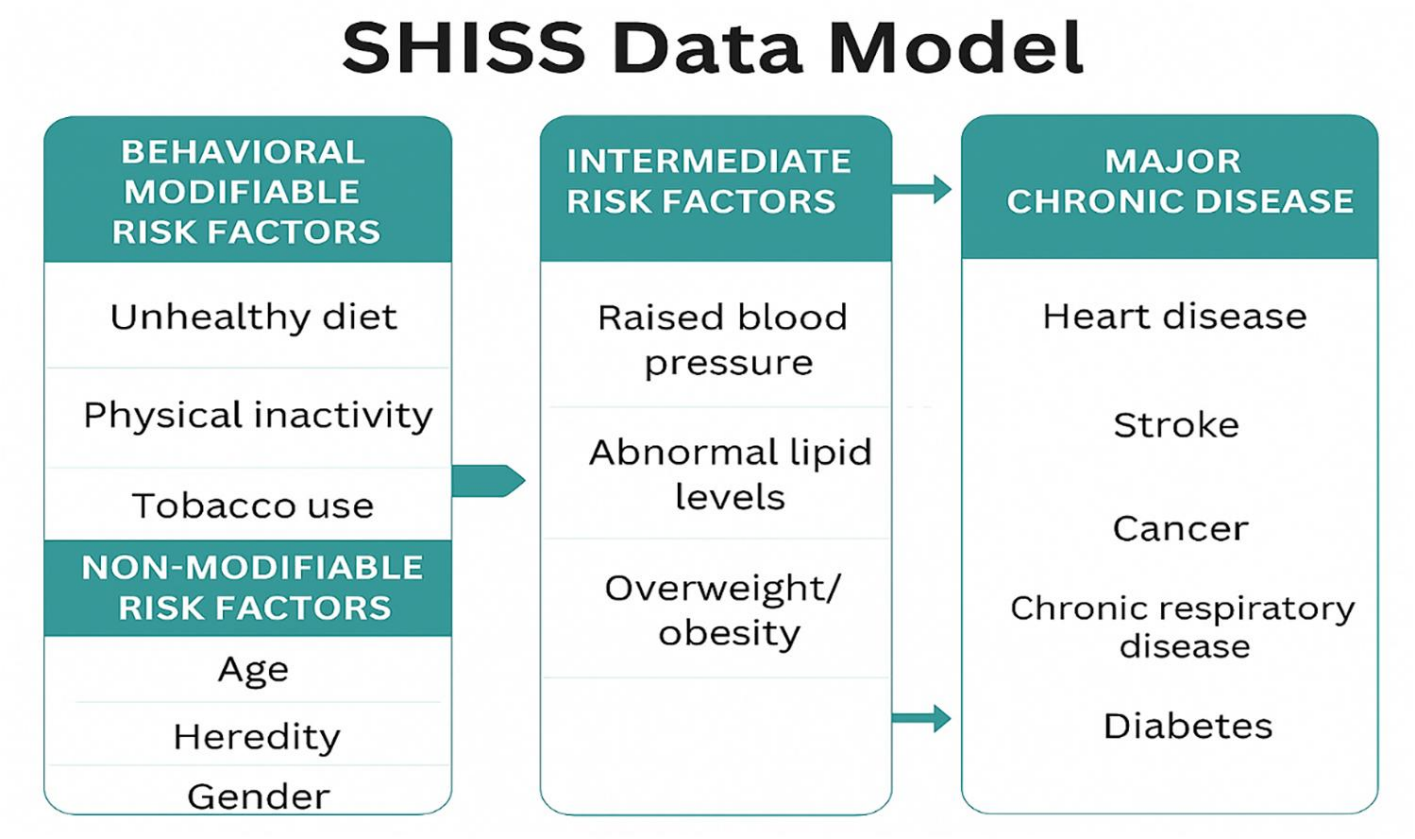
Sharik health indicators surveillance system (SHISS) data model (19). Adapted from “The scientific model for the community health indicator”, Sharek Health Research Association (https://en.sharikhealth.com/).

After providing verbal consent, participants were asked their age to determine eligibility (15). The data collector then recorded the participant's age, gender, and region in to the system to further assess quota eligibility. Subsequently, information on major chronic diseases and their major behavioral and intermediate risk factors, as suggested by the WHO and CDC, was collected (15). As shown in the SHISS data model (Figure 1), the dataset includes behavioral risk factors (diet, physical activity, and tobacco use, including cigarettes, waterpipes, and e-cigarettes), diagnosed on-treatment intermediate risk factors (HTN and hypercholesterolemia), and obesity measured by body mass index (BMI) using height and weight (15). It also includes diagnosed major chronic diseases for which participants were currently receiving treatment, such as diabetes, heart disease, stroke, cancer, and chronic respiratory disease (15, 16). Additionally, the presence of diagnosed genetic diseases was recorded as a non-modifiable risk factor. In addition, SHISS collect income, education level, and depression screening using the Patient Health Questionnaire-2 (PHQ-2) (15). Participants were asked to provide their height in centimeters and weight in kilograms, from which their body mass index (BMI) was calculated using the following formula (14):

$$\mathrm{BMI}=\left(\mathrm{Weights}(\mathrm{kg})\right)/\left(\mathrm{Heigh}t^{2}\right)$$

We used the Centers for Disease Control and Prevention (CDC) BMI category status (Underweight: Below 18.5, Normal or Healthy Weight: 18.5–24.9, Overweight: 25.0–29.9, Obese: 30.0 and Above). This study followed the WHO's global recommendations on physical activity for adults (18–64 years old): (1) vigorous-intensity physical activity (VIPA), 75 min per week, or (2) moderate-intensity physical activity (MIPA), 150 min per week. Based on participants’ self-reported responses to the interview questionnaire (i.e., number of exercise minutes, frequency, and intensity level per week), two categorical outcome variables were created to reflect whether the guidelines were met: an acceptable level of physical activity (ALPA) (at least 150 min of MIPA per week and/or at least 75 min of VIPA per week) and a low level of physical activity (LLPA) (less than 150 min of MIPA and/or less than 75 min of VIPA) (15).

Regarding diet, participants were inquired about their daily consumption of fruits and vegetables. Those whose daily intake included at least one serving of fruit and one serving of vegetables were classified as having an acceptable level of fruit and vegetable intake (AFVI). Otherwise, they were categorized as having a low level of fruit and vegetable intake (LFVI). For depression screening, the PHQ-2 was employed, with a cut-off score of 3 or higher used to identify potential cases (15).

### 2.6 Statistical analysis

Descriptive statistics were used to report prevalence. For quantitative variables with a normal distribution, the mean and standard deviation (SD) are presented; otherwise, the median and range are used. Categorical variables are shown as percentages and compared using the Pearson Chi-squared test. Multivariable logistic regression was applied to identify factors associated with hypertension. Age was entered as a continuous variable, and the odds ratio represents the relative change in odds of hypertension per 1-year increase in age among adults aged ≥18 years. Categorical predictors included gender, education, monthly income, cigarette smoking, BMI category, physical activity level, fruit/vegetable intake, depression risk, and comorbid conditions (diabetes, hypercholesterolemia, heart disease, stroke, cancer, chronic respiratory disease, and genetic disease). For multi-category variables, the reference categories were: bachelor's degree or above (education), no stable monthly income (income), never smoker (cigarette smoking), and underweight (BMI). For binary variables, the reference category was the absence of the risk factor or comorbid condition. The initial model included all covariates, followed by backward elimination to remove non-significant predictors. Age and gender were retained regardless of significance due to clinical importance. Prior to regression, multicollinearity was assessed using variance inflation factors (VIFs); all were <2.5, indicating acceptable independence among variables. Although the SHISS design inherently achieves demographic balance across age, gender, and region strata, no additional post-stratification weighting was applied, as proportional quota sampling already approximates the national population structure. Missing data were minimal (<2% for any variable) and were handled using listwise deletion. Sensitivity analyses excluding missingness confirmed consistent results. All analyses were performed using SPSS Statistics version 27.0. All analyses were performed using SPSS Statistics version 27.0 (IBM Corp., Armonk, NY), and findings were reported following the STROBE guidelines for cross-sectional studies (20).

## 3 Results

A total of 20,492 potential participants were contacted, of whom 14,007 completed the interview, yielding a 68.3% response rate across all 13 administrative regions of Saudi Arabia. Among participants, 50.1% were male and the mean age was 36.7 years (SD 13.7). The distribution of demographic characteristics by hypertension status is shown in Table 1.

**Table 1:** Participant sociodemographic in the sample by consistency hypertension status.

Variables	Hypertension status, *n* (%)		Total, *n* (%)	*P* Value ChiSquare
	Yes	No		
Sex				<.001
Male	932 (13.3)	6,087 (86.7)	7,019 (50.1)	
Female	790 (11.3)	6,198 (88.7)	6,988 (49.9)	
Age Groups (Year)				<.001
18–19	5 (0.7)	704 (99.3)	709 (5.1)	
20–29	119 (2.5)	4,553 (97.5)	4,672 (33.4)	
30–39	188 (6.7)	2,621 (93.3)	2,809 (20.1)	
40–49	460 (14.6)	2,690 (85.4)	3,150 (22.5)	
50–59	513 (29.6)	1,220 (70.4)	1,733 (12.4)	
60+	437 (46.8)	497 (53.2)	934 (6.7)	
Regions				.184
Asir	138 (12.9)	942 (87.2)	1,080 (7.7)	
Baha	115 (10.6)	967 (89.4)	1,082 (7.7)	
Eastern region	133 (12.3)	949 (87.7)	1,082 (7.7)	
Hail	133 (12.3)	950 (87.7)	1,083 (7.7)	
Jazan	141 (13.0)	943 (86.9)	1,084 (7.7)	
Al Jouf	133 (12.3)	948 (87.7)	1,081 (7.7)	
Madinah	134 (12.8)	913 (87.2)	1,047 (7.5)	
Makkah	159 (14.7)	923 (85.3)	1,082 (7.7)	
Najran	109 (10.4)	938 (89.6)	1,047 (7.5)	
Northern border	117 (10.8)	967 (89.2)	1,084 (7.7)	
Qassim	143 (13.2)	941 (86.8)	1,084 (7.7)	
Riyadh	136 (12.5)	953 (87.5)	1,089 (7.8)	
Tabuk	131 (12.1)	951 (87.9)	1,082 (7.7)	
Education				<.001
Less than bachelor's degree	1,121 (15.9)	5,922 (84.1)	7,043 (50.3)	
bachelor's degree or above	601 (8.6)	6,363 (91.4)	6,964 (49.7)	
Monthly Income				<.001
No stable monthly Income	144 (9.9)	1,307 (90.1)	1,451 (10.4)	
Less than 5,000 SAR^a^	497 (8.9)	5,105 (91.1)	5,602 (40.0)	
5,001 to 11,000 SAR	516 (14.1)	3,147 (85.9)	3,663 (26.2)	
More than 11,000 SAR	565 (17.1)	2,726 (82.9)	3,291 (23.4)	
Grand Total	1,722	12,374	14,007	

a SAR, Saudi Arabian Riyal.

Among all respondents, 1,722 participants reported hypertension, giving an overall prevalence of 12.3%. Of these, 54.1% were male, and 92.8% were aged 30 years or older. The highest proportion (32.8%) reported a monthly income above 11,000 SAR. Regarding behavioral factors, 92.3% had low fruit and vegetable intake (LFVI), 82.5% reported low physical activity (LLPA), and 22.8% were cigarette smokers.

Participants with hypertension also showed high rates of comorbidities: hypercholesterolemia (47.9%), diabetes (42.8%), overweight or obesity (75.2%), heart disease (20.2%), stroke (5.8%), cancer (4.1%), chronic respiratory disease (12.3%), genetic disorders (13.2%), and an increased risk of depression (23.8%) (Tables 1, 2).

**Table 2:** Prevalence of chronic diseases and risk factors in the study participants (*n* = 14,007) by hypertension status.

Variables	Hypertension status		Total, *n* (%)	*P* Value ChiSquare
	Yes	No		
Fruit and Vegetable Intake				<.001
AFVI	132 (20.9)	499 (79.1)	631 (4.5)	
LFVI	1,590 (11.9)	11,786 (88.1)	13,376 (95.5)	
Physical Activity				.084
ALPA	301 (11.3)	2,362 (88.7)	2,663 (19.0)	
LLPA	1,421 (12.5)	9,923 (87.5)	11,344 (81.0)	
Cigarette Smoking				<.001
Never	1,329 (11.6)	10,164 (88.4)	11,493 (82.1)	
Yes, daily	251 (15.1)	1,410 (84.9)	1,661 (11.9)	
Yes, occasionally	142 (16.6)	711 (83.4)	853 (6.1)	
Waterpipe Smoking				.040
Never	1,468 (12.1)	10,739 (87.9)	12,207 (87.1)	
Yes, daily	91 (14.4)	539 (85.6)	630 (4.5)	
Yes, occasionally	163 (13.9)	1,007 (86.1)	1,170 (8.4)	
E-Cigarette Smoking				.805
Never	1,578 (12.3)	11,223 (87.7)	12,801 (91.4)	
Yes, daily	64 (12.5)	447 (87.5)	511 (3.6)	
Yes, occasionally	80 (11.5)	615 (88.5)	695 (5.0)	
Hypercholesterolemia				<.001
Yes	824 (49.3)	849 (50.7)	1,722 (12.3)	
No	898 (7.3)	11,436 (92.7)	12,285 (87.7)	
Diabetes				<.001
Yes	737 (45.1)	896 (54.9)	1,673 (11.9)	
No	985 (7.9)	11,389 (92.1)	12,334 (88.1)	
BMI Category				<.001
Underweight	22 (2.9)	734 (97.1)	756 (5.4)	
Normal	405 (7.1)	5,274 (92.9)	5,679 (40.5)	
Overweight	646 (14.3)	3,870 (85.7)	4,516 (32.2)	
Obese	649 (21.2)	2,407 (78.8)	3,056 (21.8)	
Heart disease				<.001
Yes	347 (58.2)	249 (41.8)	596 (4.3)	
No	1,375 (10.3)	12,036 (89.7)	13,411 (95.7)	
Stroke				<.001
Yes	100 (59.2)	69 (40.8)	169 (1.2)	
No	1,622 (11.7)	12,216 (88.3)	13,838 (98.8)	
Cancer				<.001
Yes	71 (39.2)	110 (60.8)	181 (1.3)	
No	1,651 (11.9)	12,175 (88.1)	13,826 (98.7)	
Chronic Respiratory Disease				<.001
Yes	211 (22.1)	744 (77.9)	955 (6.8)	
No	1,511 (11.6)	11,541 (88.4)	13,052 (93.2)	
Genetic Diseases				<.001
Yes	227 (32.1)	480 (67.9)	707 (5.0)	
No	1,495 (11.2)	11,805 (88.8)	13,300 (95.0)	
Risk of Depression				<.001
Yes	409 (15.5)	2,227 (84.5)	2,636 (18.8)	
No	1,313 (11.6)	10,058 (88.4)	11,371 (81.2)	

AFVI, Acceptable Fruit and Vegetable Intake; LFVI, Low Fruit and Vegetable Intake; ALPA, Acceptable Level of Physical Activity; LLPA, Low Level of Physical Activity; BMI, Body Mass Index; HTN, Hypertension.

### 3.1 Logistic regression analysis

Table 3 summarizes the multivariable logistic regression model for factors associated with hypertension. In the multivariable logistic regression model (Table 3), age was modeled per 1-year increase among adults aged ≥18 years. Increasing age (AOR = 1.06; 95% CI 1.05–1.06; *p* < 0.001), education below bachelor's degree (AOR = 1.31; 95% CI 1.14–1.50; *p* < 0.001), daily or occasional smoking (*p* < 0.05), and overweight or obesity (AOR = 1.93 and 2.53, respectively; *p* < 0.01) were significantly associated with higher odds of hypertension. Reference categories for the categorical predictors are shown explicitly in Table 3 and include: bachelor's degree or above (education), no stable monthly income (income), never smoker (cigarette smoking), underweight (BMI), and absence of each comorbid condition or risk factor.

**Table 3:** Multivariable logistic regression model for factors associated with hypertension among adults in Saudi Arabia (2021).

Variable	Category	Odds ratio (95% Confidence Interval)	*P* value
Age	Per 1-year increase	**1.06** **(1.05–1.06)**	<.001
Education	Bachelor's degree or above (reference)	1.00 (reference)	–
	Less than bachelor's degree	**1.31** **(1.14–1.50)**	<.001
Monthly income	No stable monthly income (reference)	1.00 (reference)	–
	<5,000 SAR	0.80 (0.63–1.00)	.053
	5,001–11,000 SAR	0.98 (0.78–1.24)	.888
	>11,000 SAR	0.97 (0.76–1.23)	.793
Risk of depression	No (reference)	1.00 (reference)	–
	Yes	**1.40** **(1.20–1.63)**	<.001
Cigarette smoking	Never (reference)	1.00 (reference)	–
	Yes, daily	**1.23** **(1.03–1.48)**	.024
	Yes, occasionally	**1.64** **(1.28–2.08)**	<.001
Diabetes	No (reference)	1.00 (reference)	–
	Yes	**2.64** **(2.28–3.05)**	<.001
Hypercholesterolemia	No (reference)	1.00 (reference)	–
	Yes	**4.09** **(3.56–4.71)**	<.001
Genetic diseases	No (reference)	1.00 (reference)	–
	Yes	**1.62** **(1.30–2.03)**	<.001
Stroke	No (reference)	1.00 (reference)	–
	Yes	**2.22** **(1.48–3.33)**	<.001
Cancer	No (reference)	1.00 (reference)	–
	Yes	**1.84** **(1.26–2.68)**	.002
Chronic respiratory disease	No (reference)	1.00 (reference)	–
	Yes	**1.35** **(1.09–1.66)**	.006
Heart disease	No (reference)	1.00 (reference)	–
	Yes	**3.54** **(2.84–4.42)**	<.001
BMI category	Underweight (reference)	1.00 (reference)	–
	Normal	1.36 (0.85–2.20)	.204
	Overweight	**1.93** **(1.20–3.11)**	.007
	Obese	**2.53** **(1.57–4.08)**	<.001

AOR, Adjusted odds ratio; CI, Confidence interval; SAR, Saudi Riyal; BMI, Body mass index.

Age was entered as a continuous variable and the odds ratio represents the change in odds of hypertension per 1-year increase in age among adults aged ≥18 years.

For binary variables (e.g., diabetes, hypercholesterolemia, heart disease), the reference category is the absence of the condition.

For multi-category variables (education, monthly income, cigarette smoking, BMI), the explicitly labeled “reference” category has OR = 1.00 and serves as the baseline for comparison.

Bold values indicate statistically significant results (*p* < 0.05).

Participants with diabetes (AOR = 2.64), hypercholesterolemia (AOR = 4.09), and heart disease (AOR = 3.54) had markedly greater odds of hypertension (all *p* < 0.001). Elevated odds were also observed for those reporting stroke, cancer, chronic respiratory disease, genetic conditions, and depression risk (*p* < 0.05 for all).

Reference categories are shown in Table 3 with odds ratios fixed at 1.00 and were not interpreted as risk factors themselves; all reported odds ratios for other categories are relative to these reference groups.

### 3.2 Model diagnostics

The logistic regression model demonstrated adequate goodness-of-fit (Hosmer–Lemeshow *χ*² = 7.84, *p* = 0.45) and acceptable explanatory power (Nagelkerke R² = 0.41). The model's discriminatory ability was strong, with an area under the ROC curve (AUC) = 0.87 (95% CI 0.85–0.88), indicating excellent distinction between hypertensive and non-hypertensive participants. No evidence of multicollinearity was detected (all VIFs < 2.5). All *p*-values in tables are reported using standard conventions (<0.05, < 0.01, < 0.001), and confidence intervals are provided to support interpretation of effect sizes.

## 4 Discussion

The results of this nationwide study on HTN in Saudi Arabia provide a comprehensive view of the prevalence and associated comorbidities among adults. With a response rate of 68.3%, the study included 14,007 participants, of whom 12.2% were diagnosed with HTN. This estimate is lower than the pooled national prevalence of 22.66% reported by Alshammari et al. (2023) (4). This discrepancy is expected, as our case definition was limited to physician-diagnosed and currently treated individuals excluding undiagnosed or untreated cases and our sample skewed younger (mean age 36.7 years) (1, 4, 6, 12). Prior probability-based surveys using measured blood pressure have shown higher rates due to inclusion of unrecognized hypertension. Thus, the present estimate more accurately reflects the “aware and managed” segment of the hypertensive population rather than the total biological prevalence. Beyond the percentage itself, the pattern that matters for practice is clustering of HTN with modifiable risks (tobacco, excess weight) and cardio metabolic multimorbidity (diabetes, hypercholesterolemia

Consistent with a national survey by Alenazi and Alqahtani (2023), HTN prevalence ranged from 6% to 10% across regions, with markedly higher rates observed in older age groups (40). Our findings corroborate these trends, with HTN prevalence rising sharply with age particularly after age 50 and being slightly more common among males. While regional variation exists, our uniform quota across all 13 regions may attenuate detectable differences; programmatically, age-related risk remains the dominant stratifier.

The regression model identified several significant factors associated with HTN. Higher age, lower education levels, and the presence of chronic diseases such as diabetes, hypercholesterolemia, genetic diseases, stroke, cancer, chronic respiratory disease, and heart disease were all linked to living with HTN. These associations are consistent with global and regional literature (2, 8, 14, 15) and highlight two practice levers: (1) an education gradient suggesting equity-focused tailoring of risk communication and access; and (2) multimorbidity clustering that favors bundled, rather than siloed, chronic-disease management. For example, individuals with diabetes had more than double the odds of HTN, consistent with the metabolic syndrome clustering described in other Middle Eastern studies (13). Behavioral factors (daily and occasional smoking) were significant, reinforcing that cardio metabolic and behavioral risks co-occur and should be addressed together (2, 9).

Tobacco exposure aligns with vascular mechanisms linking smoking to elevated BP (9, 10); waterpipe and e-cigarette patterns merit surveillance given rising regional uptake. Excess weight (overweight/obesity) showed strong associations, echoing NCD-RisC and Saudi evidence (8, 17); embedding structured weight-management support alongside BP care is therefore warranted (17).

Participants with HTN were also found to have a higher prevalence of other chronic diseases, exacerbating the risk of severe health complications. For instance, hypertensive individuals with diabetes or hypercholesterolemia are at a significantly increased risk of cardiovascular events such as myocardial infarctions and strokes. The combination of these conditions can lead to more complex medical management and poorer health outcomes, emphasizing the need for integrated care approaches (10, 12). Moreover, obesity, identified in a significant proportion of hypertensive participants, further aggravates these risks due to its association with increased blood pressure and other metabolic disorders (8). Practically, these patterns argue for single-visit bundled care (BP, lipids, glucose, tobacco, weight) to reduce fragmentation and loss to follow-up.

For population health, three complementary approaches follow: (i) detection—normalize opportunistic BP checks in PHC and community pharmacies with automated prompts for high-risk profiles (older age, lower education, diabetes/dyslipidemia); (ii) integrated management—deliver an NCD bundle that co-manages BP, lipids, glucose, weight, and tobacco with shared registries and team-based follow-up; and (iii) behavior change at scale—brief tobacco interventions with referral, standardized weight-management pathways, and culturally adapted messaging (6).

In addition, public health policies that influence population behavior and reduce expo-sure to dietary risk factors are crucial for strategically reducing HTN prevalence and complications in the larger population. For example, the Saudi Food and Drug Authority (SFDA) has launched various health policies that may contribute to better chronic disease control in the future, including a ban on trans fats in all processed foods and voluntary agreements with food manufacturers to reduce salt and sugar content in processed food products (21–23). Continued monitoring should link such policies to intermediate outcomes (salt/sugar content, BP screening coverage) and clinical control rates to inform iterative improvement.

Strengths include the large sample (*n* = 14,007), nationwide coverage across all 13 regions, and standardized instrument enabling internally coherent associations consistent with external literature. These features support generalizable implications for service design while acknowledging the measurement constraints noted. Enhanced HTN management and systematic screening for associated risk factors are essential to improving patient outcomes. Integrating comprehensive screening programs into primary healthcare services can facilitate early identification and management of HTN, especially among high-risk populations. Regular monitoring and follow-up care are critical to effectively managing blood pressure and associated comorbidities. Training healthcare providers on the latest HTN management guidelines and encouraging adherence to these guidelines can further improve patient outcomes. Studies have demonstrated that structured HTN management, including pharmacological treatment and lifestyle interventions, significantly reduces the risk of cardiovascular events and enhances the quality of life for patients (6, 8). By addressing the multifaceted risk factors and comorbid conditions associated with HTN through public health initiatives and improved clinical practices, it is possible to reduce the burden of HTN and enhance the overall health of the Saudi population.

The study has some limitations that should be considered when interpreting the findings. Firstly, the cross-sectional design of the study restricts the ability to establish causal relationships between HTN and associated risk factors or comorbidities. Secondly, the reliance on self-reported data for HTN status, comorbid conditions, and behavioral risk factors may introduce response bias and inaccuracies. Additionally, because the survey was conducted via telephone interviews, some population groups, such as individuals without reliable phone access or those less likely to participate in health surveys, may have been underrepresented, introducing potential noncoverage bias, in addition to the sample was restricted to Arabic-speaking Saudi residents, which may limit the generalizability of the findings to other populations. Third, our on-treatment diagnosis case definition (self-reported diagnosis plus current treatment) likely underestimates true HTN prevalence by excluding undiagnosed/untreated cases; as a result, comparisons with measurement-based surveys should be made cautiously. Fourth, Self-reported height and weight are known to underestimate obesity prevalence, which may attenuate the observed association between body mass index and hypertension. Fifth, the quota sampling strategy ensured balanced representation by age, gender, and region, it differs from probability sampling and may limit strict generalizability, as individuals more health-aware or responsive by phone could be overrepresented. Nevertheless, the automated quota controls and large coverage mitigate major sampling biases and maintain internal validity.

From a policy perspective, the findings of this study underscore the urgent need for a comprehensive and integrated national strategy to strengthen hypertension detection, management, and prevention across the Saudi health system. The identification of strong multimorbidity clusters particularly the coexistence of hypertension with diabetes, dyslipidemia, obesity, and tobacco use highlights the necessity of shifting from disease-specific programs toward bundled chronic-disease care pathways within primary health care. Integrating hypertension management with diabetes and cardiovascular risk control, supported by shared electronic registries and multidisciplinary care teams, could substantially improve clinical outcomes and reduce service fragmentation. Moreover, scaling up opportunistic blood-pressure screening in primary health centers, community pharmacies, and workplaces would help identify undiagnosed cases early, especially among high-risk groups such as older adults and individuals with limited education. Behavioral interventions also remain a critical pillar; embedding brief tobacco cessation counseling and structured weight-management services into routine care can complement broader population-level efforts led by the Saudi Food and Drug Authority (SFDA) to reduce salt, sugar, and trans-fat consumption. Collectively, these initiatives align with the Health Sector Transformation Program and Adaa performance indicators, advancing the objectives of Saudi Vision 2030 by promoting equitable, preventive, and data-driven models of care that reduce cardiovascular morbidity and enhance population well-being.

## 5 Conclusions

This study provides an updated national profile of hypertension among adults in Saudi Arabia, revealing a prevalence of 12.3% and highlighting its strong associations with older age, lower educational attainment, excess weight, smoking, diabetes, and hypercholesterolemia. The clustering of hypertension with these behavioral and metabolic risk factors underscores the complexity of multimorbidity patterns within the Saudi population. These findings contribute valuable insights to ongoing national surveillance efforts and support the design of integrated strategies for noncommunicable disease prevention and control. To our knowledge, this is one of the first studies to use the 2021 SHISS dataset to comprehensively examine sociodemographic, behavioral, and clinical factors associated with hypertension in Saudi Arabia.

## Data Availability

The original contributions presented in the study are included in the article/Supplementary Material, further inquiries can be directed to the corresponding author/s.
